# Modeling of demoralization in breast cancer

**DOI:** 10.3389/fpsyt.2025.1554128

**Published:** 2025-04-25

**Authors:** John M. de Figueiredo, Robert Kohn, Joyce Y. Chung, Sara Gostoli

**Affiliations:** ^1^ School of Medicine, Yale University, New Haven, CT, United States; ^2^ School of Public Health, Brown University, Providence, RI, United States; ^3^ Therapeutic Radiology, School of Medicine, Yale University, New Haven, CT, United States; ^4^ Department of Psychology "Renzo Canestrari", University of Bologna, Bologna, Italy

**Keywords:** demoralization, breast cancer, oncology, depression, subjective incompetence, helplessness, hopelessness

## Abstract

**Objective:**

This study aimed at identifying correlates of demoralization in breast cancer.

**Materials and methods:**

Information was obtained from outpatients with breast cancer at the Oncology Clinic of a university-affiliated hospital in the United States, using reliable and valid scales, and from the participants’ medical records on demographic and social characteristics, location, type, and stage of cancer, whether it was a re-occurrence or first time diagnosed, type of cancer treatment, medications being used, history of mental disorder, functional impairment, perceived stress, perceived social support, resilience, subjective incompetence, demoralization, and depression. Demoralization was measured with the Demoralization Scale. Bivariable and multivariable analyses were conducted with demoralization as the dependent variable.

**Results:**

Demoralization correlated positively with functional impairment, perceived stress, depression, and subjective incompetence, and negatively with months since diagnosed with breast cancer, perceived social support, resilience, and quality of life. Forward stepwise regression conducted without depression in the regression equation identified emotional wellbeing subscale of quality of life, resilience, subjective incompetence, perceived social support, and functional impairment as significant. After forced entry of depression, perceived social support and mild depression ceased to be significant, leaving only moderate and severe depression as significant. Perceived stress did not enter any of the regression models.

**Conclusion:**

Early detection of demoralization and of the co-occurrence of depression and demoralization are essential for promoting the well-being of patients with breast cancer. Psychotherapy should focus on strengthening the modifiable negative correlates of demoralization, countering the modifiable positive correlates, and preventing the co-occurrence of demoralization and depression.

## Introduction

Prevalence rates for depression in cancer patients vary significantly, with reported figures ranging from 0% to 38% for major depressive disorder and 3% to 31% for depression spectrum syndromes. Specifically, in breast cancer, the prevalence of depressed mood is estimated to be between 1.5% and 46% (1). These wide variations are likely due to differences in research methodologies.

Recently, medical literature has focused on demoralization, a treatable condition that differs from depression spectrum disorders, although it may occur alongside them. Frank introduced the term “demoralization” in psychiatric discussions, defining it as a state of mind characterized by a loss of spirit or courage, feelings of disheartenment, confusion, and disorder (2). He suggested that this mindset is common among many outpatients seeking psychotherapy, regardless of their specific diagnostic labels.

Although several definitions have been proposed, a consensus has emerged that demoralization is a psychological state characterized by subjective incompetence and symptoms of distress, such as depression, anxiety, resentment, anger, loss of meaning or purpose in life, or combinations thereof. Subjective incompetence is a self-perceived incapacity to perform tasks and express feelings deemed appropriate in a stressful situation, resulting in pervasive uncertainty and doubts about the future. Subjective incompetence has been described as a clinical hallmark of demoralization and interpreted as a loss of the cognitive map to deal with a stressful situation (3). If unrecognized or untreated, subjective incompetence embedded in demoralization may progress to helplessness, hopelessness, a sense of failure, irrelevance, or futility, and eventually to demands for hastened death or suicide (4).

Taking demoralization into account in assessments enhances the evaluation of the clinical complexity (5). Toward this goal, diagnostic criteria have been proposed and a scale of subjective incompetence and several scales of demoralization have been developed (6–11). Two clinical interviews are also available (12, 13). The application of these criteria and scales made it possible to recognize demoralization in a variety of clinical settings, physical illnesses, and mental disorders, in victims of stressful situations, such as refugees, and in the general population (14, 15).

Depression and demoralization can occur independently, but they have distinct clinical characteristics and require different interventions. In cases of demoralization, individuals are willing to take action to improve their situation, but they may feel uncertain about how to proceed. In contrast, individuals experiencing depression often have a reduced willingness to act, even if they are aware of potential solutions. This uncertainty associated with demoralization reflects a sense of subjective incompetence (16).

Demoralization has been observed in 13% to 18% of patients with progressive diseases, including cancer (17, 18). In a study involving 105 breast cancer patients, the most frequently identified psychosomatic syndromes, according to the Diagnostic Criteria for Psychosomatic Research (DCPR), were demoralization, health anxiety, and alexithymia. Patients experiencing demoralization were more likely to feel hopeless, have a lower quality of life, and exhibit greater worries and preoccupations related to their cancer. They also tended to use less effective coping strategies, often adopting a pessimistic and helpless attitude toward their condition (19). A follow-up study of early-stage breast cancer patients examined the impact of helplessness, hopelessness, depression, and fighting spirit on outcomes. It found that helplessness and hopelessness continued to adversely affect 10-year disease-free survival, while depression did not have the same negative impact (20). Additionally, a study of 142 breast cancer patients in Italy revealed that 24.6% met the criteria for DCPR demoralization. This condition was associated with lower perceived social support and reduced quality of life (21, 22).

The roles of subjective incompetence and its opposite, resilience, have been studied in the context of breast cancer. Research indicates that resilience is inversely related to demoralization, subjective incompetence, depression, and quality of life (23). Specifically, subjective incompetence in breast cancer patients is directly linked to perceived stress and inversely to perceived social support (24). After primary treatment—such as surgery, chemotherapy, radiation therapy, and/or targeted therapy—demoralization can hinder the interrelations of stress, sleep disturbances, and psychological well-being. This effect is particularly significant during the first five years following a breast cancer diagnosis (25). Furthermore, demoralization in breast cancer patients is associated with a lower quality of life, poorer sleep quality, and reduced spiritual interest. Patients who experience both depression and demoralization tend to have the worst prognosis (26).

Demoralization has been observed in patients with breast cancer, but the factors that contribute to it are not fully understood. Identifying these factors and understanding their relative impact on demoralization could help in early diagnosis and in targeting preventive and therapeutic interventions. The objective of this study was to identify the correlates of demoralization in breast cancer patients.

## Materials and methods

Before any data were collected, approval of the Yale University Institutional Review Board was obtained, potential participants were told by the Principal Investigator that the study aimed to assess mental state and quality of life, and written informed consent from the participants was obtained. This research was conducted ethically in accordance with the World Medical Association Declaration of Helsinki.

### Design and participants

This was an observational study with a cross-sectional design. Participants were recruited from a consecutive sample of outpatients with breast cancer at the Oncology Clinic of a university-affiliated hospital in the United States. The following were the admission criteria: biopsy confirmed breast cancer as the primary diagnosis; age 20 to 90 years; female; and able to read and understand English. Age 20 was used as the lower age limit because breast cancer in women younger than 20 is very rare and may present unique psychosocial challenges (27). Individuals admitted to hospice were excluded.

### Data sources and assessments

All participants were evaluated and diagnosed by a board certified oncologist (JC). Participants completed a questionnaire eliciting information on sociodemographic characteristics (date of birth, gender, race, marital status, education, and income). Medical and psychiatric history was obtained from medical records by trained research assistants and included information about the location and type of cancer (primary or metastatic), stage, whether it was a first-time diagnosis or a re-occurrence, type of cancer treatment (surgery, chemotherapy, or radiation therapy), and the medications being used (Tamoxifen, Arimidex, antidepressants, benzodiazepines, other). Research assistants asked the participants and recorded if there was a past psychiatric history, The following scales were used:

- Demoralization Scale (DS), a psychometric measure that treats demoralization as a continuous variable and consists of items rated on a 5-point scale ranging from 0 (never) to 4 (all the time), with four subscales, loss of meaning and purpose, disheartenment, dysphoria, and sense of failure, and a total score calculated by summarizing the single subscale scores with higher scores indicating higher levels of demoralization (8).
- Subjective Incompetence Scale (SIS), a 12-item scale designed to evaluate themanifestations of subjective incompetence in the past two weeks. For each item, the response alternatives are ranked on a 4-point Likert scale ranging from 0 (none of the time) to 3 (most or all of the time), reflecting the frequency of the corresponding experience (e.g. “puzzled, indecisive, and uncertain as to what actions to take”). The sum score is calculated, yielding a range from 0 to 36 with higher scores indicating greater levels of subjective incompetence (9, 10).
- Beck Depression Inventory (BDI), a 21-item, multiple-choice inventory with four response choices according to the severity of the symptoms during the past week, ranging from the absence of a symptom (0) to maximum severity (3) (28).
- Eastern Cooperative Oncology Group Performance Status Rating Scale (ECOG) to assess functional impairment. This is a rating scale that measures how the disease impacts a patient’s daily living abilities with 5 levels of increasing impairment ranging from “fully active, able to carry on all pre-disease performance without restriction” (0) to “being dead” (5) (29).
- Impact of Event Scale (IES) to assess perceived stress linked to having cancer. This is a 15-item measure of perceived stress resulting from trauma across 2 domains, avoidance and intrusion, with frequency of “not at all, rarely, sometimes, often” over the past 7 days (30).
- Connor Davidson Resilience Scale (CD-RISC) to assess resilience. This scale comprises 25 items rated on a 5-point scale (0-4), with higher scores reflecting greater resilience (31).
- Interpersonal Support Evaluation List, Short Form (ISEL-SF) to assess perceived social support. This is a series of 15 statements about perceived social support, each rated as “completely false, somewhat false, somewhat true, and completely true.” (32).
- RAND 36 Item Health Survey (SF-36), version 1.0, to assess quality of life. As part of the Medical Outcomes Study (MOS), a multi-site, multi-year study to explain variations in patient outcomes, RAND Corporation developed this 36-item survey of quality of life across eight health dimensions: physical functioning, bodily pain, role limitations due to physical health problems, role limitations due to personal or emotional problems, emotional well-being, social functioning, energy/fatigue, and general health perceptions. It also includes a single item that provides an indication of perceived change in health. Pre-coded numeric values are coded according to a scoring key. A high score defines a more favorable health state. Then, the original response is recoded on a 0 to 100 range. Each score is the percentage of total possible score achieved. Then, items in the same scale are averaged together to create the 8 scale scores (33, 34).

All scales were self-administered, except ECOG that was administered by the oncologist. All scales have been widely used in research and have been shown to have adequate psychometric properties in the original articles describing the scales (8–10, 28–34).

### Statistical analysis

#### Sample size calculation

G*Power software (latest version, 3.1.9.7; Heinrich-Heine-Universität Düsseldorf, Düsseldorf, Germany; http://www.gpower.hhu.de/) was used to calculate the sample size (35). Assuming a medium effect size (Cohen’s f^2^ ≥ 0.15), a sample size of 130 would yield a power of 0.9 in multiple linear regression with 1 predictor at a two-tailed α level of 0.05. Assuming a rejection rate of 40%, it was determined that the number of participants meeting the admission criteria would have to be about 200 to 250.

#### Data analysis

Data were analyzed using SPSS 24.0 (IBM Corp., Armonk, NY 2016). All available data were used. Items with missing data were not removed because there were very few such items, and imputation was not done for the same reason. For all tests performed, significance level was set *a priori* at p < 0.05 (two-tailed).

Frequency distributions of the variables were obtained. Univariable analyses were conducted to find the means and standard deviations of all variables. Bivariable associations between all study variables and the DS score were studied using t-tests, one way analysis of variance, and Pearson correlations. Depression as measured by the BDI score was classified into 3 levels: no depression or minimal (BDI: 0 - 9); mild (BDI: 10 - 18); moderate (BDI: 19 - 29); and severe (BDI: 30 - 63) (28). “Moderate” and “severe” categories were merged due to sample sizes of each being small.

A forward stepwise regression model was obtained with demoralization as measured by DS as a dependent variable and those variables found to be statistically significant in the bivariate analysis (except depression as measured by BDI) as independent variables. Depression was then forced into the regression equation as a categorical variable to estimate the improvement in R^2^ produced by the addition of the BDI score and to calculate its statistical significance for each level of depression.

## Results

### Characteristics of the sample

A total of 243 patients met the admission criteria and were invited to participate. Of these, 92 declined (“not interested”), giving a participation rate of 62%. There were no significant differences in the age distributions between those who declined and the 151 who participated.

Participants (N = 151) were female with an age range of 30 to 87 (mean age: 63.7 ± 10.7). The majority were white (83.4%, N = 126). A minority were married (49.7%, N = 75), had more than a high school education (38.4%, N = 58), with a third having a monthly household income range of $2001 to $6000 (N = 50). The mean number of months since the diagnosis of cancer was 45. ± 40.9. The type of cancer was primary (nonmetastatic) in 95.9% (N = 140), metastatic in 4.1% (N = 6), and unknown in 3.3% (N = 5). In 90.7% (N = 137) it was diagnosed for the first time. Cancer stages were distributed as follows: stage 1: 20.5%, N = 31; stage 2: 42.4%, N = 64; stage 3:16.6%, N = 25; stage 4: 4.6%, N = 7; and unknown: 16%, N = 24. There was no re-occurrence in 74.2% (N = 118), recurrence in 13.9% (N = 21), and it was unknown in 7.9% (N = 12). Only 24 participants (15.9%) had a psychiatric diagnosis.

Treatments for cancer included radiation in 136 participants (90.1%), surgery in 132 (87.4%), and chemotherapy in 62 (41.1%). Tamoxifen was prescribed to 13 participants (8.6%) and Anastrozole to 15 (9.9%). There were 20 participants (13.2%) on antidepressants and 12 (7.9%) on benzodiazepines.

### Univariable analyses

Results of univariable analyses are given in **Table 1**. In addition, BDI scores were distributed as follows: 112 (74.2%) had no or minimal depression; 31 (20.5%) had mild depression; and 8 (5.3%) had moderate or severe depression.

**Table 1:** Univariable analysis.

Variable	Scale	N	Mean	SD
Demoralization	DS	151	15.2	13.2
Subjective incompetence	SIS	151	6.2	7.27
Impairment	ECOG	150	0.3	0.49
Perceived stress total	IES Total	150	21.6	18.1
Perceived stress-avoidance	IES Avoidance	149	12.0	10.9
Perceived stress intrusion	IES Intrusion	149	9.5	9.4
Resilience	CD-RISC	151	81.8	14.6
Perceived social support	ISEL-SF	150	39.7	7.4
QOL Physical functioning	SF-36 Physical functioning	149	72.9	25.2
QOL Bodily pain	SF-36 Bodily pain	149	74.7	26.7
QOL Emotional role limitations	SF-36 Emotional role limitations	149	80.3	36.4
QOL Energy and fatigue	SF-36 Energy an fatigue	149	55.3	25.2
QOL Emotional wellbeing	SF-36 Emotional wellbeing	149	77.4	20.3
QOL Social functioning	SF-36 Social functioning	149	84.1	22.8
QOL General health perceptions	SF-36 General health perceptions	149	69.3	22.1
QOL Health change	SF-36 Health change	149	53.2	25.2
QOL Composite	SF-36 Composite	149	71.1	20.1

DS, Demoralization scale SIS; Subjective incompetence scale; ECOG, Eastern Cooperative Oncology Group Performance Status Rating Scale; IES, Impact of Event Scale; CD-RISC, Connor Davidson Resilience Scale; ISEL-SF, Interpersonal Support Evaluation List, Short Form; QOL, Quality of Life, SF-36, Short-Form 36 Health Survey.

### Bivariable analyses

Statistically significant positive correlations with demoralization were found for the following variables: functional impairment, perceived stress (total, avoidance, and intrusive), depression, and subjective incompetence. Significant negative correlations were found for months since diagnosed with breast cancer, perceived social support, resilience, and quality of life (**Table 2**).

**Table 2:** Pearson correlations with demoralization.

Variable	Scale	N	r	p <
Months since diagnosed with breast cancer		139	-0.190	0.025
Functional impairment	ECOG	150	0.280	0.001
Perceived stress	IES	150	0.345	0.001
Perceived stress avoidance	IES	149	0.247	0.002
Perceived stress intrusion	IES	149	0.356	0.001
Perceived social support	ISEL-SF	150	-0.591	0.001
Depression	BDI	151	0.794	0.001
Subjective incompetence	SIS	151	0.659	0.001
Resilience	CD-RISC	151	-0.727	0.001
QOL: Physical health	SF-36	149	-0.332	0.001
QOL: Limitation health	SF-36	149	-0.382	0.001
QOL: Limitation emotion	SF-36	149	-0.564	0.001
QOL: Energy/fatigue	SF-36	149	-0.499	0.001
QOL: Emotional well-being	SF-36	149	-0.744	0.001
QOL: Social function	SF-36	149	-0.609	0.001
QOL: Pain	SF-36	149	-0.345	0.001
QOL: General health	SF-36	149	-0.388	0.001
QOL: Health change	SF-36	149	-0.230	0.005

N, Number of participants; r, Pearson product moment correlation coefficient; p, Two tailed probability (significance); QOL, Quality of Life as measured by SF-36; ECOG, Eastern Cooperative Oncology Group Performance Status Assessment; IES, Impact of Events Scale. ISEL-SF, Interpersonal Support Evaluation List Short Form; BDI, Beck Depression Inventory (with depression treated as a continuous variable); SIS, Subjective Incompetence Scale; CD-RISC, Connor Davidson Resilience Scale; SF-36, 36-Item Short Form Survey.

### Multivariable analysis

Forward stepwise regression modeling without depression identified the following succession of variables: emotional well-being subscale of quality of life, resilience, subjective incompetence, perceived social support, and functional impairment (**Table 3**). After the forced entry of categorical depression, perceived social support ceased to be significant and mild depression was not significant, leaving only moderate and severe depression as significant (**Table 4**). Notably, perceived stress did not enter any of the regression models.

**Table 3:** Forward regression with demoralization as the dependent variable without depression in the model.

Model	R	R^2^	Adj R^2^	SEE	R2 C	FC	df	P(FC) <	AIC	R
	0.871	0.758	0.749	6.530	0.009	5.333	1,14	0.022	561.296	0.871
Model	SS		df		MS		F		p <	
Regression	18947.648		5		3789.50		88.86		0.001	
Residual	6055.183		142		3789.50					
Total	25002.831		147							
	B		SE		β		t		p <	
(Constant)	67.144		5.578				14.231		0.001	
Wellbeing (SF 36)	-0.242		0.035		-0.377		-6.889		0.001	
Resilience (CD-RISC)	-0.329		0.050		-0.358		-6.597		0.001	
Subjective incompetence (SIS)	0.317		0.098		0.177		3.232		0.002	
Perceived social support (ISEL-SF)	-0.231		0.095		0.130		-2.430		0.016	
Functional impairment (ECOG)	2.673		1.158		0.099		2.309		0.022	

Adj R^2^, Adjusted R^2^; SEE, Standard error of estimate; R^2^C, R^2^change; FC, F change; p(FC), significance of F change; AIC, Akaike information criterion; SS, Sum of squares; df, Degrees of freedom; MS, Mean of squares; p, Probability (significance); b, Unstandardized coefficient; SE, Standard error; β (Beta), Standardized coefficient; SF-36, 36-Item Short Form Survey; CD-RISC, Connor Davidson Resilience Scale; SIS, Subjective Incompetence Scale; ISEL-SF, Interpersonal Support Evaluation List Short Form; ECOG, Eastern Cooperative Oncology Group Performance Status Assessment.

**Table 4:** Forward regression with demoralization as the dependent variable with depression in the model.

Model	R	R^2^	Adj R^2^	SEE	R2 C	FC	df	P(FC) <	AIC	R
	0.884	0.781	0.770	6.258	0.023	7.301	2,14	0.001	550.612	0.884
Model	SS		df		MS		F		p <	
Regression	19519.577		7		2788.511		71.197		0.001	
Residual	5483.254		140		3789.50					
Total	25002.831		147		39.166					
	B		SE		β		t		p <	
(Constant)	63.921		5.102				12.529		0.001	
Wellbeing (SF 36)	-0.236		0.035		-0.368		-6.794		0.001	
Resilience (CD-RISC)	-0.344		0.049		-0.375		-6.973		0.001	
Subjective incompetence (SIS)	0.245		0.099		0.137		2.488		0.014	
Perceived social support (ISEL-SF)	-0.124		0.096		-0.070		1.297		0.197	
Functional impairment (ECOG)	2.688		1.144		0.100		2.350		0.020	
Depression, mild (BDI)	-1.370		1.611		-0.42		-0.851		0.396	
Depression, moderate + severe (BDI)	9.334		3.117		0.162		2.995		0.003	

Adj R^2^, Adjusted R^2^; SEE, Standard error of estimate; R^2^C, R^2^change; FC, F change; p(FC), significance of F change; AIC, Akaike information criterion; SS, Sum of squares; df, Degrees of freedom; MS, Mean of squares; F, F statistic; p, Probability (significance); b, Unstandardized coefficient; SE, Standard error; β (Beta), Standardized coefficient; t, t statistic; SF 36, SF-36, 36-Item Short Form Survey; CD-RISC, Connor Davidson Resilience Scale; SIS, Subjective Incompetence Scale; ISEL-SF, Interpersonal Support Evaluation List Short Form; ECOG, Eastern Cooperative Oncology Group Performance Status Assessment; BDI, Beck Depression Inventory.

## Discussion

Ambulatory patients with breast cancer were assessed for levels of demoralization, depression, and sociodemographic, clinical, and treatment-related variables. Regression analysis showed that emotional well-being, resilience, subjective incompetence, perceived social support, and functional impairment are significantly associated with demoralization when depression is not considered. When depression was included in the analysis, perceived social support and mild depression were no longer significant, while moderate and severe depression remained significant.

The findings enhance our understanding of demoralization in breast cancer by highlighting emotional well-being, resilience, and perceived social support as positive correlates, while subjective incompetence and functional impairment are identified as negative correlates of demoralization. Perceived stress lost its statistical significance when other variables are considered, potentially because it became “normalized” over time as the disease progresses. This is supported by the significant negative correlation observed between the severity of demoralization and the number of months since a breast cancer diagnosis. Furthermore, as depression severity increases, the importance of perceived social support diminishes, possibly due to a decline in patients’ confidence in their supportive resources.

The results support the hypothesis that a greater severity of depression is linked to the cooccurrence of demoralization and depression. This complements the findings of previous studies which found that increasing levels of demoralization are associated with a higher degree of depression (36). This conclusion has important implications for diagnosis, treatment, and prognosis.

The results highlight the need to differentiate between depression and demoralization in comprehensive clinical interviews of patients with breast cancer. Anhedonia is a symptom of a major depressive episode, but absent in demoralization, and subjective incompetence, helplessness, and hopelessness point to demoralization, not depression. It should be noted that the most recent edition of the Diagnostic and Statistical Manual of Mental Disorders (DSM-5TR) (37) does not include subjective incompetence, helplessness, or hopelessness as criteria for a major depressive episode. Lack of concentration, anergia, insomnia, and anorexia are usually absent in demoralization but may be present in depressive spectrum disorders. Demoralization always occurs in the context of a past, present, anticipated, or imagined stressful situation (predicament) and is more likely when the stressful situation is relevant to the person’s self-esteem. Depression may or may not be precipitated by a stressful life event (15). Most studies have found that demoralization is correlated with lower income, higher perceived stress, lower perceived social support, lower resilience, higher levels of functional impairment, and lower quality of life (16–26). The clinical interview should inquire about well-being, resilience, subjective incompetence, helplessness, hopelessness, possible “normalization” of perceived stress, illness denial, confidence in perceived social support, and functional impairment, as well as anhedonia and suicidal ideation, intent, and plan. This is particularly important in patients with cancer given the observation that the presence of core depressive symptoms is very low in cancer patients diagnosed with depressive disorder (38).

Two case examples illustrate how the findings of this research can be applied in clinical practice. Both cases involve adult women who were in their usual state of good health until they discovered a lump in their breasts during self-examination. They promptly discussed their findings with their primary care physicians, who obtained a detailed medical history, conducted physical examinations, and ordered mammograms. The mammograms suggested malignancy, leading to biopsies that confirmed the diagnosis of breast cancer. Subsequently, both women were referred to oncologists who evaluated their conditions and proposed treatment plans that included surgery and chemotherapy. After discussing their treatment options with their oncologists, both women felt increasingly discouraged. Both were highly respected professionals, one a researcher, the other a teacher. Past personal and family histories for mental disorders or alcohol and substance use were negative for both.

Their clinical trajectories diverged from that point on. One woman struggled to envision a rewarding career in research following her diagnosis. Although she managed to focus on her work and maintained a good appetite and sleep, she expressed to her primary care physician that even though her husband and children were supportive, and enjoyed doing fun things with them, she felt lost about how her future would unfold. This sense of an “uncertain future” and lack of meaning and purpose in life intensified with time and caused her considerable distress. She frequently asked herself: “what is the point of living like this?.” A few months later, she felt powerless to change her situation and believed that her fate was sealed. The thought of a hastened death had crossed her mind but she categorically denied suicidal ideation, intent, or plan. Her oncologist recognized that she was demoralized but not clinically depressed. Instead of prescribing antidepressants, he referred her to a psychiatrist for psychotherapy. As a result, her mood improved significantly, allowing her to cope better with her illness and changing circumstances. Knowing of the close interrelations of demoralization and depression, the psychiatrist continued to monitor her mental status for symptoms of depressive disorder, such as insomnia, anorexia, anergia, and anhedonia.

In contrast, the other woman, despite also having a supportive family, began to experience increased sadness following her diagnosis. She started losing interest in things she once enjoyed, had a reduced appetite, and suffered from both initial insomnia and early morning awakening. She found it difficult to concentrate on her work and felt increasingly weak. After ruling out that her symptoms were caused by her physical condition or chemotherapy, her oncologist recommended that she consult a psychiatrist. She declined, thinking that her symptoms were understandable given her diagnosis of cancer. Over time, she developed feelings similar to those experienced by the first woman. She felt overwhelmed by her cancer, powerless to change her circumstances, and saw no end to her suffering. At that point she decided to follow her oncologist’s advice and consulted a psychiatrist. She reported to the psychiatrist her symptoms and stated that the wish to die had also come to her mind, but she, too, denied suicidal ideation, intent, or plan. The psychiatrist diagnosed her with a major depressive disorder and prescribed an antidepressant. While her appetite. sleep, and energy improved, and her anhedonia decreased, she continued to experience feelings of subjective incompetence, loss of meaning and purpose in life, helplessness, and hopelessness. The psychiatrist recognized these residual symptoms as manifestations of demoralization and the need to intervene before her condition escalated to existential despair. The treatment plan was adjusted to include psychotherapy. This resulted in a more realistic and accurate appraisal of her situation and significant improvement in her mood.

Early detection and reversal of demoralization and depression are crucial to prevent the co-occurrence of both. Longitudinal use of the clinimetric method, as exemplified by the Diagnostic Criteria for Psychosomatic Research (DCPR), would probably identify certain unique aspects of individual profiles associated with this co-occurrence that may not be immediately obvious from the use of psychometric measures (39). Previous research has shown that in cancer patients, demoralization, but not depression, is associated with a significantly increased risk for suicidal ideation after controlling for all mental disorders (40–42). Given this evidence, future research should determine if the co-occurrence of demoralization and depression escalates the risk of suicidal ideation. The results agree with previous findings that demoralization is associated with lower quality of life and that the combination of demoralization and depression worsens the prognosis (18–25).

Psychotherapy aims at countering demoralization and should focus on reducing subjective incompetence and functional impairment as well as strengthening well-being and resilience. If possible, confidence on perceived social support should also be strengthened. Together with evidence from previous studies, the results support the continuing need for psychotherapy with increasing severity of demoralization and depression (43). There is agreement that the appropriate intervention for demoralization is “a selection of a range of cognitively informed, existentially oriented, and meaning-centered psychotherapies” (44). Psychotherapeutic interventions have been developed specifically tailored to reduce demoralization by modifying the perception of stress, restoring hope, and replacing negative cognitive distortions of self and stressful situations with positive and more precise and realistic appraisals (45). Examples of proposed interventions are meaning-centered psychotherapy (46, 47), sequential combination of cognitive-behavioral psychotherapy and well-being psychotherapy (48), psilocybin-assisted psychotherapy (49), and supportive psychotherapy at the bedside (50). Future research should examine the impact of the co-occurrence of demoralization and depression on adherence to treatment and on medical outcomes as well as the efficacy of psychotherapy at preventing and treating the co-occurrence of demoralization and depression with increasing severity of both. The efficacy of antidepressants at countering the co-occurrence of demoralization and depression should be further investigated. The results are consistent with previous reports suggesting that the magnitude of the benefit of antidepressants increases with the severity of depression (51) and that in major depressive disorder, a greater degree of hopelessness significantly increases the risk for non-response to antidepressant treatment and the risk of greater endpoint depression severity after controlling for depression severity at baseline (52).

The limitations of this study should be recognized. This was a cross-sectional study with a one-time assessment and no follow-up observations. Participants were outpatients at a single academic hospital, thereby limiting generalizations to patients in similar centers. The cross-sectional design precludes causal inferences. Certain potential correlates of demoralization, such as anxiety, allostatic overload, and mental pain, were not examined. The scales used, while reliable and valid, had different time frames. Ideally, assessments should have included both clinician-rated and self-reported measures (53), but at the time of this study, clinician-rated versions of the self-administered scales employed were unavailable. The use of a clinimetric scale, such as the Diagnostic Criteria for Psychosomatic Research Demoralization scale (DCPRD), might have strengthened the conclusions (7). Although consecutive admissions were invited to join the study and an effort was made to avoid bias in sample selection and data collection, bias may have occurred favoring the selection of participants with milder depression over the more severely depressed patients who might have refused to participate. This is suggested by the limited number of participants in each of moderate and severe depression groups. A longitudinal study of a random sample of patients with diverse sociocultural backgrounds stratified for varying levels of depression, including the more severe ones, and evaluated by psychiatrists or psychologists with both clinimetric and psychometric methods would complement our findings and further clarify the course of demoralization and its relationship to increasing depression and vice-versa.

The study also has strengths. Participants were all evaluated, diagnosed, and rated for functional impairment by a Board certified oncologist. Reliable, valid, sensitive, and specific scales were used to assess a wide range of demographic, clinical, psychological, and treatment related variables.

## Conclusions

In this study of ambulatory patients with breast cancer, a co-occurrence of demoralization and depression with increasing severity of depression was documented. Subjective incompetence, functional impairment, lower resilience, lower sense of wellbeing, and lower perceived social support correlated with the severity of demoralization. Perceived stress lost its statistical significance when other variables were considered. With increasing severity of depression, perceived social support also lost its statistical significance. These results have implications for the diagnosis and reversal of demoralization in breast cancer.

## Data Availability

The raw data supporting the conclusions of this article can be found here: https://doi.org/10.60600/YU/DU3K6F.
